# Thrombin generation during a regular menstrual cycle in women with von Willebrand disease

**DOI:** 10.1038/s41598-018-35897-0

**Published:** 2018-11-30

**Authors:** Igor Govorov, Katarina Bremme, Tomas L. Lindahl, Margareta Holmström, Eduard Komlichenko, Roza Chaireti, Miriam Mints

**Affiliations:** 1 Department of Women’s and Children’s Health, Karolinska Institutet, Stockholm, Sweden; 2 Department of Clinical and Experimental Medicine, Linköping University, Linköping, Sweden; 3 Department of Medicine, Karolinska University Hospital Solna, Stockholm, Sweden; 4 Institution of Pediatrics and Perinatology, Almazov National Medical Research Centre, Saint-Petersburg, Russia; 5 Department of Molecular Medicine and Surgery, Karolinska Institutet, Stockholm, Sweden

## Abstract

Fluctuations of the sex steroids during the menstrual cycle might significantly influence hemostasis. This association, derived from a number of the observations on healthy women, is yet to be described in females affected by bleeding disorders. The aim of the current study was to assess the changes in hemostatic variables in women with vWD during two phases of the menstrual cycle (follicular and luteal) and to compare it with healthy controls. The study group included 12 vWD-affected females with regular menstrual cycle, with none of them being prescribed any hormonal treatment. The control group consisted of 102 healthy females, matched for age and BMI. Within the vWD group FVIII and FX were both significantly higher during follicular phase than in luteal phase (p = 0.013 and p = 0.033 respectively). AT, FII, FVII and FX were higher in women with vWD, compared with controls during both phases of the menstrual cycle (p < 0.0005, p < 0.0005, p = 0.001 and p < 0.0005). In women with vWD, lag time and time to peak were prolonged during both phases of the menstrual cycle(p < 0.0005), while peak thrombin concentration was reduced (p = 0.003 and p = 0.002 during follicular and luteal phase respectively) compared to healthy peers. Lower levels of FVIII and FX during luteal phase may predispose women to the development of the menorrhagia - common complication of vWD. Women with vWD need more time to reach the peak thrombin concentration, while the latter still remains less than in healthy women. Higher levels of AT in vWD-affected females, compared to controls, may also contribute to the existing bleeding tendency in this cohort.

## Introduction

Precise assessment of hemostasis is of great clinical importance, especially among patients with suspected bleeding disorders, such as von Willebrand disease (vWD). vWD is the most common inherited bleeding disorder with an estimate prevalence reaching 1% of general population^1,2^. vWD is classified based on whether a quantitative or qualitative defect in von Willebrand factor (vWF) is present, with type 1 and type 3 vWD representing reduced or almost absent vWF:Ag respectively, while four subtypes of vWD type 2 (2A, 2B, 2M or 2N) occur due to the specific dysfunction of vWF^3^. Males and females are considered to be equally affected, though clinical manifestations are more pronounced in women due to the specific hemostatic challenges during childbirth and menstruation^4^.

At the same time, accurate measurement of hemostatic variables is challenging for a number of reasons. Firstly, there are significant inter- and intra-individual variations in the levels of coagulation factors and at least some of them are dependent on a variety of physiological conditions (stress, exercise, etc.)^5,6^.

One of the most explicit examples of the hemostasis sensitivity is the changes in coagulation variables as a response to cyclic fluctuations of the sex hormones in women during normal menstrual cycle^7,8^.

All this together leads to the fact that at the moment it is rather difficult to deduce the robust patterns of how do hemostatic variables change during menstrual cycle^8^. In an attempt to increase our confidence in evaluating hemostasis, so-called global assays are used, for instance, thrombin generation test. Thrombin generation assay was proposed by Hemker and is designed to avoid the drawbacks of the conventional coagulation tests, providing a more detailed picture of hemostasis^9^.

Understanding of how does hemostasis change during the menstrual cycle is important not only for choosing the right time for analysis, but also for studying the fundamental mechanisms of Willebrand disease.

In the current study, we aim to characterize changes in hemostasis during menstrual cycle in women with vWD by measuring different hemostatic variables together with global hemostatic assay - thrombin generation in order to expand knowledge on the reasons for excessive menstrual bleeding in the majority of women with vWD.

## Results

The study group included 12 female patients aged 35,0 (33,0–41,0), hereinafter [median (25–75 percentiles)], with BMI = 23,1 (20,2–29,4). vWD types distribution was as follows: type 1–7 patients, type 2–3 patients (with 2 of them having subtype 2 M), unspecified - 2 patients. Most of them have used antifibrinolytics previously in life, although not at the time of blood sampling.

Firstly, we assessed levels of the hemostatic variables in follicular and luteal phase in women with von Willebrand disease. The results are presented as median (25–75 percentiles) and summarized in Table 1. FVIII and FX were both significantly higher during follicular phase than in luteal phase (p = 0,013 and p = 0,033 respectively). None of the thrombin generation parameters differed between the menstrual cycle phases within the vWD group.

**Table 1:** Hemostatic variables during menstrual cycle in women with von Willebrand disease.

	Follicular phase (cd 2–5)	Luteal phase (cd 22–25)	P
Fibrinogen, g/l	2.74 (2.37–2.92)	2.63 (2.28–3.02)	0.260
D-dimer, mg/l	0.06 (0.03–0.11)	0.04 (0.02–0.08)	0.124
Antithrombin, kIU/l	1.07 (1.02–1.12)	1.07 (1.01–1.17)	0.970
FII, kIU/l	1.13 (1.06–1.21)	1.15 (1.12–1.20)	0.875
FVII, kIU/l	1.11 (0.90–1.36)	1.12 (0.87–1.20)	0.240
FVIII,kIU/l	0.87 (0.52–1.14)	0.76 (0.49–1.12)	**0**.**013**
FX, kIU/l	1.08 (1.03–1.30)	1.04 (0.98–1.29)	**0**.**033**
vWF, kIU/l	0.68 (0.21–1.04)	0.65 (0.20–1.02)	0.577
**Thrombin generation parameters**			
Lag-time, min	3.22 (2.87–3.33)	3.00 (2.71–3.30)	0.679
ETP, nM*min	1698.36 (1450.22–2021.40)	1863.57 (1417.09–2113.60)	0.083
Peak, nM	215.00 (170.61–300.16)	242.02 (194.88–295.91)	0.240
Ttpeak, min	7.33 (6.44–7.89)	6.95 (6.33–7.22)	0.520

p < 0,05 (statistical significance) is marked in bold.

kIU/l, kilo International Units.

When comparing hemostatic variables between patients with vWD and healthy controls following differences were observed. In follicular phase AT, FII, FVII and FX were higher in women with vWD, compared with controls. VWF levels were expectedly lower in the study group, although the p-value appeared to be slightly above the established boundary for statistical significance (p = 0,059).

During luteal phase, the abovementioned differences persisted. In addition, vWF was now significantly lower in patients with vWD than in controls (p = 0,044). D-dimer levels were also lower in vWD group, which had not been observed during follicular phase.

Table 2 illustrates the comparison of the levels of the hemostatic components during follicular phase and luteal phases between the groups.

**Table 2:** Comparison of hemostatic variables during follicular (cd 2–5) and luteal (cd 22–25) phases between vWD-affected females and healthy controls.

	Follicular phase			Luteal phase		
	vWD patients	Healthy controls	P	vWD patients	Healthy controls	P
Fibrinogen, g/l	2.74 (2.37–2.92)	2.58 (2.34–2.84)	0.522	2.63 (2.28–3.02)	2.67 (2.42–2.97)	0.552
D-dimer, mg/l	0.06 (0.03–0.11)	0.06 (0.04–0.09)	0.545	0.04 (0.02–0.08)	0.06 (0.04–0.09)	**0**.**032**
Antithrombin, kIU/l	1.07 (1.02–1.12)	0.98 (0.93–1.04)	**<0**.**0005**	1.07 (1.01–1.17)	0.97 (0.93–1.03)	**<0**.**0005**
FII, kIU/l	1.13 (1.06–1.21)	0.99 (0.94–1.04)	**<0**.**0005**	1.15 (1.12–1.20)	0.99 (0.94–1.04)	**<0**.**0005**
FVII, kIU/l	1.11 (0.90–1.36)	0.87 (0.76–0.99)	**0**.**001**	1.12 (0.87–1.20)	0.82 (0.73–0.93)	**0**.**001**
FVIII, kIU/l	0.87 (0.52–1.14)	0.92 (0.78–1.09)	0.446	0.76 (0.49–1.12)	0.97 (0.83–1.08)	0.074
FX, kIU/l	1.08 (1.03–1.30)	0.95 (0.86–1.02)	**<0**.**0005**	1.04 (0.98–1.29)	0.91 (0.85–1.01)	**<0**.**0005**
vWF, kIU/l	0.68 (0.21–1.04)	0.80 (0.63–1.17)	0.059	0.65 (0.20–1.02)	0.86 (0.62–1.05)	**0**.**044**

p < 0,05 (statistical significance) is marked in bold.

Regarding thrombin generation parameters, lag-time and time to peak were significantly prolonged in vWD-affected females (in all cases p < 0,0005), while peak thrombin concentration was lower (p < 0,003). Interestingly, ETP, depicting the total amount of generated thrombin, did not differ significantly between the groups.

**Figure 1:**
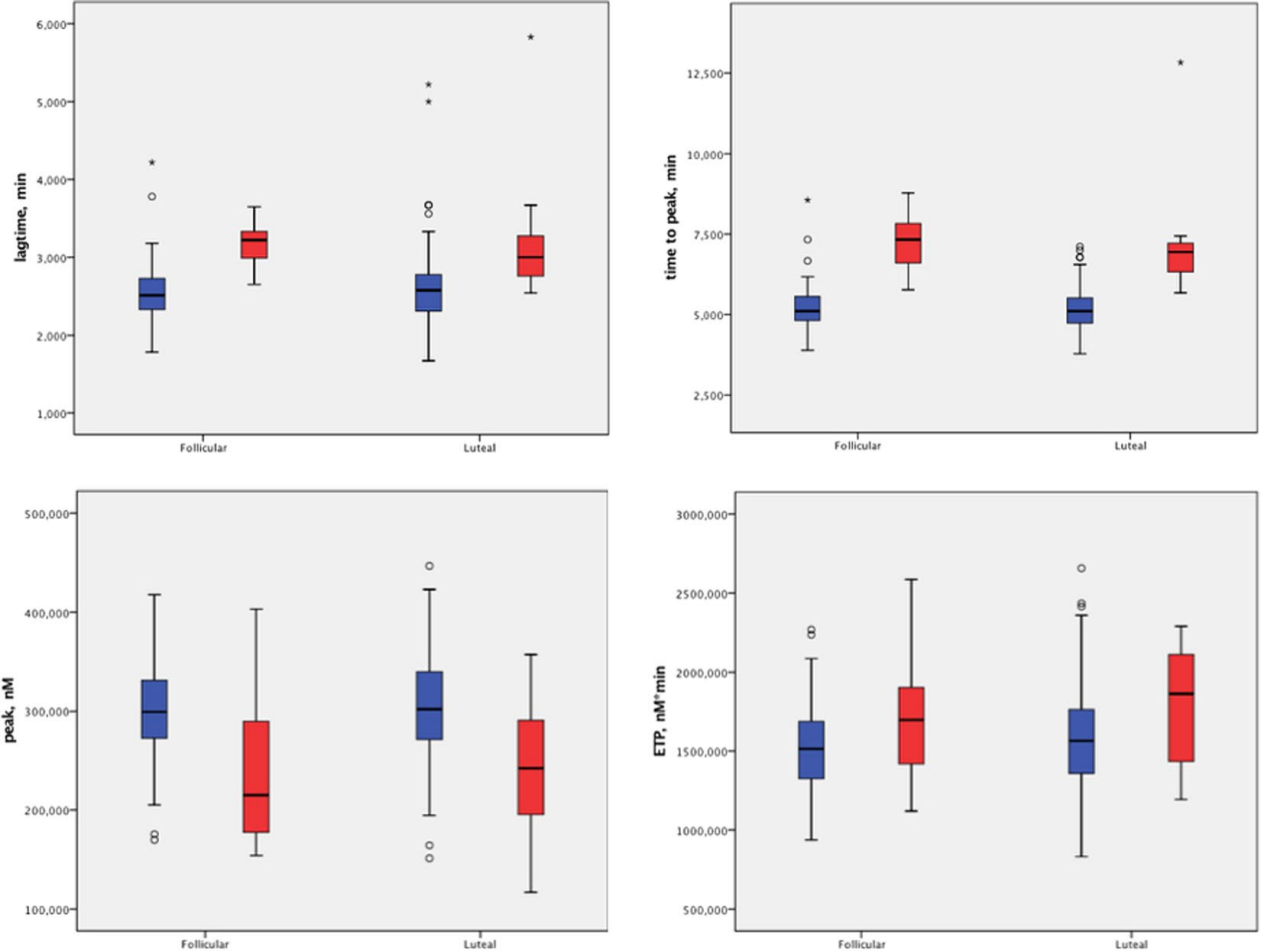
Comparison of the thrombin generation parameters between the groups during two phases of the menstrual cycle. vWD patients are marked in , while healthy controls in .

During the luteal phase, the thrombin generation pattern resembled that during the follicular phase, with lag-time, time to peak being prolonged and peak height diminished. Endogenous thrombin potential was comparable between the groups. Figure 1 depicts the differences in thrombin generation parameters between the groups during two phases of the menstrual cycle.

## Discussion

In the current study we found lower levels of factor VIII and X during luteal phase compared to the follicular phase in women with vWD. In addition, in women with vWD we observed altered thrombin generation pattern with prolonged lag time, time to peak and reduced peak thrombin concentration, although the overall ETP was equal to healthy controls. Interestingly, factors II, VII and X were higher in women with vWD, compared to the control group. To the best of our knowledge, hemostatic variables including thrombin generation profiles during menstrual cycle have not yet been studied in women with vWD.

Blood clotting system sensitively responds to the minor changes in homeostasis, *i*.*a*. cyclic fluctuations of the female sex steroids during menstrual cycle. This phenomenon has been the subject of many studies, albeit the ultimate results are often contradictory^8,12–15^. At the same time, a thorough description of how does exactly hemostasis react to the hormonal variations is not only essential to choose a proper timing for coagulation analyses within the menstrual cycle, but also may provide valuable information about fundamental mechanisms underlying hematological disorders. Furthermore, it helps to optimize treatment options.

In women with vWD FVIII and FX appeared to be higher during follicular phase, compared to the healthy controls. The latter was also demonstrated in the study by Chaireti *et al*. on healthy volunteers^10^. Regarding FVIII, the majority of the previous studies found no pronounced cyclic variations in its levels^7,14,16–20^, while others reported lowest FVIII during menstruation or early follicular phase^21,22^. Since FVIII and FX are both procoagulant agents and interact intimately, the decrease in their levels during the luteal phase may predispose women to heavy menstrual bleeding - the common complication of vWD^23^.

Other variables, including the thrombin generation markers did not differ significantly in women with vWD, which is in contrast to the healthy control group, where ETP was higher during luteal phase. Nevertheless, it is difficult to confidently state whether there is actually no difference or the number of observations in the study group has not been large enough to demonstrate it.

More pronounced differences were found when comparing the levels of hemostatic parameters between vWD patients and control group during two phases of the menstrual cycle.

While assessing the thrombin generation, significant differences were observed in the majority of the markers. Lag-time and time to peak were prolonged in women with vWD, compared to controls during both phases of the menstrual cycle (p < 0,0005 for all calculations). Peak thrombin concentration was lower in follicular and luteal phase in women with vWD (p = 0,003 and p = 0,002 respectively). At the same time, the total amount of generated thrombin (ETP) did not differ between the groups in either phase. Such a flattened thrombin generation curve in vWD patients is consistent with previous studies^11^ and suggests that in order to achieve effective hemostasis, it might be more important to reach maximum thrombin concentrations in a shorter time than to generate somewhat normal total thrombin amount over a longer period. However, this observation should optimally be corroborated by exact clinical data. In general, the thrombin generation test, often used today to evaluate the global hemostatic potential, in our study has reaffirmed its applicability in differentiating patients with vWD and controls.

Interestingly, AT was higher in women with vWD both during follicular and luteal phases (p < 0,0005). AT suppresses coagulation through inhibiting several serine proteases, in particular thrombin^24^. Thus, we hypothesize that increased activity of AT might be one of the mechanisms contributing to reduced thrombin generation in women with vWD and thus play role in development of the bleeding complications.

FII, FVII and FX were higher in women with vWD, than in controls (p < 0,0005; p = 0,01; p < 0,0005 respectively). This observation is not clearly understood, but since the groups were similar in terms of age and BMI, probably some other factors might play role. This may include, but not be limited to the well-known modifiers of the hemostasis, such as, smoking status or blood group, or may reflect activation of the coagulation cascade to compensate for the bleeding^25^.

D-dimer differed significantly between vWD-affected females and healthy controls only during luteal phase, being lower in the former (p = 0,032). The previous studies reported antagonistic results on the levels of D-dimer during menstrual cycle and therefore it is rather difficult to deduce any consistent pattern^14,26^. However, the relatively higher D-dimer levels during early follicular phase (which corresponds to menstrual phase of the uterine cycle) may reflect the activation of local fibrinolysis within the uterus.

The current study has several limitations. More frequent blood sampling might be required to provide a more detailed description. Previous studies on hemostatic variables during menstrual cycle varied considerably with respect to the exact days of blood sampling and the ultimate number of measurements, as described in the recent systematic review by Knol *et al*.^8^. Furthermore the ultimate results might depend both on preanalytical/analytical conditions and the design of a study itself (longitudinal, cross-sectional)^27^. In each case, it is, of course, necessary to balance the scope of the study with the patient’s risks associated with the relative invasiveness of the procedure. Another drawback is that the control for blood-group of study participants has not been performed. Blood group glycoproteins are intimately intertwined with vWF and it has been shown that in people with 0-blood group vWF and FVIII are decreased in comparison with non-0 blood groups^28,29^. At the same time, it does not explain the differences in other coagulation variables. Lack of patients with type 3 vWD may be considered as a limitation of study, as it limits how representative our cohort is, thus requiring the larger studies. However, the relative rarity of these patients presents certain difficulties in recruiting. On the other hand, the majority of the patients with vWD have type I, which means that the results are applicable in the majority of women with vWD refereed and treated because of menstrual bleeding.

Stringent inclusion criteria may be regarded as the strength of the study. None of the women were on the hormonal treatment, although normally vWD females are often prescribed with them, in order to control excessive menstrual bleeding.

To summarize, the differences in the levels of hemostatic parameters between vWD patients and healthy controls were significant but at the same time remained largely the same whether being assessed during follicular or luteal phase of the menstrual cycle. This implies, that regardless of the timepoint of blood sampling, the coagulation tests may provide valuable information, especially if a global hemostasis assay, such as thrombin generation is employed.

To conclude, in women with vWD the levels of coagulation factors FVIII and FX are lower during the luteal phase of the menstrual cycle, which may contribute to developing excessive menstrual bleeding. Compared to healthy women, the time to reach the peak thrombin concentration was prolonged in the women with vWD, with the latter being still lower than in healthy women. This occurs despite an increase in the levels of the procoagulants: FII, FVII and FX. Probably, the existing malfunction of hemostasis in patients with vWD depends not only on the qualitative or quantitative defect of vWF, but also on the increase in antithrombin levels.

## Materials and Methods

This was a longitudinal study, measuring hemostatic variables on the same group of women during two phases of menstrual cycle.

The inclusion criteria were as follows: vWD-affected female patients, age limits 18–52, with regular menstrual cycle (21–35 days), with none of them being prescribed with medications, containing hormones (combined oral contraceptives, contraceptive implants, intrauterine devices, HRT). In all patients vWD was diagnosed based on the conventional criteria: bleeding tendency, family history positive for prolonged bleedings and low vWF. In some patients additional tests were performed, such as vWF multimer assessment. Pregnant and breast-feeding women were excluded, as were those who either had irregular menstrual cycle or even none, due to physiological or iatrogenic menopause. In total 12 patients were recruited.

Blood samples were taken twice in each patient: one during follicular phase (cycle day, cd 2–5) and one during luteal phase (cd 22–25). Afterwards, the blood levels of the following components were assessed: fibrinogen, antithrombin, factors II, VII, VIII, X, von Willebrand and D-dimer, supplemented with the assessment of thrombin generation. The results were then compared with those from healthy controls (n = 102), coming from the recent study by Chaireti *et al*.^10^.

In all patients’ venous blood samples were drawn from an antecubital vein after 15 min in the supine position. All samples were drawn in the morning after an overnight fast. Blood samples for analysis of coagulation factors were collected in citrated tubes and immediately centrifuged at 2000g for 15 min. After removal of the cells, plasma was re-centrifuged for another 15 min at 2000g. Cell-free plasma (platelet poor plasma, PPP) was stored at −70 °C until analysed.

The current study is done in cooperation with Almazov National Medical Research Centre, Saint-Petersburg, Russia.

### Ethical permission

All methods were performed in accordance with the relevant guidelines and regulations.

All participants were informed with the characteristics of the study and its voluntary nature. Written informed consent was obtained from all participants. Personal data was made anonymous directly after collection. The current study was approved by Stockholm Regional Ethics Committee (№ 2016/503–31) and was subsequently supplemented with the local permission from Almazov National Medical Research Centre in Saint-Petersburg, Russia (№ 17/ПЦ).

### Measurement of hemostatic variables

Thrombin generation was measured by the calibrated automated thrombogram method as described in the Thrombogram Guide by Thrombinoscope BV (Maastricht, the Netherlands). We computed following thrombin generation parameters: lag-time (time point at which thrombin generation starts, in minutes), time to peak (time to reach max thrombin concentration, in minutes), endogenous thrombin potential (ETP, total amount of generated thrombin, in nM*min), peak (max thrombin concentration, in nM). All samples were measured in triplicate. The final mixture of PPP reagent (trigger) and PPP used in the assay contained 5 pM tissue factor (TF) and 4 µM phospholipids. All reagents were obtained from Thrombinoscope BV, Masstricht, The Netherlands. 96-well plates used were obtained from Ninolab, Stockholm, Sweden.

Clauss method was employed to measure fibrinogen. Hemostatic variables were measured by means of the Sysmex CS 2000i from Siemens Healthcare Diagnostics (Stockholm, Sweden). Antithrombin (AT) and factor VIII (FVIII) were assessed by a chromogenic methods; factors II, VII and X (FII, FVII and FX) - clotting methods, von Willebrand factor (vWF:Ag) - immunochemical method. All reagents were produced by Siemens Healthcare Diagnostics (Stockholm, Sweden). D-dimer was measured by a latex-enhanced immunochemical method, using reagents from Medirox (Studsvik, Sweden)

### Statistical analysis

Statistical analysis was performed using SPSS 24 for Mac OS. Background data between the groups was compared using unpaired two-sample t-test.

Since there are no studies with a design similar to ours, *i*.*e*. measuring hemostatic markers in vWD patients during the menstrual cycle, we calculated the required power for the cohort by using results from the studies by Chaireti *et al*.^10^ and Rugeri *et al*.^11^, where thrombin generation was measured in healthy women and patients with von Willebrand disease, respectively. The required cohort size in order to achieve a power of 0,8 with a type I-error of 5% was 12 patients.

Due to predominant non-normal distribution and relatively small number of participants in the study group, non-parametric tests were chosen for calculations. We used Wilcoxon signed-rank test to compare changes in hemostatic variables during menstrual cycle within study group. Mann-Whitney U test was employed to assess differences between control and study groups. In all cases an exact p-value was calculated, while statistical significance was set at p < 0,05.

## Data Availability

The datasets generated during and/or analysed during the current study are available from the corresponding author on reasonable request.

## References

[1] Bowman M, Hopman WM, Rapson D, Lillicrap D, James P (2010). The prevalence of symptomatic von Willebrand disease in primary care practice. J. Thromb. Haemost..

[2] Rodeghiero F, Castaman G, Dini E (1987). Epidemiological investigation of the prevalence of von Willebrand’s disease. Blood.

[3] Sadler JE (2006). Update on the pathophysiology and classification of von Willebrand disease: a report of the Subcommittee on von Willebrand Factor. J. Thromb. Haemost..

[4] James AH (2009). Von Willebrand disease in women: awareness and diagnosis. Thromb. Res..

[5] Ikarugi H (2003). High intensity exercise enhances platelet reactivity to shear stress and coagulation during and after exercise. Pathophysiol Haemost Thromb.

[6] Lippi G, Maffulli N (2009). Biological influence of physical exercise on hemostasis. Semin Thromb Hemost.

[7] Kadir RA, Economides DL, Sabin CA, Owens D, Lee CA (1999). Variations in coagulation factors in women: effects of age, ethnicity, menstrual cycle and combined oral contraceptive. Thromb. Haemost..

[8] Knol HM, Kemperman RF, Kluin-Nelemans HC, Mulder AB, Meijer K (2012). Haemostatic variables during normal menstrual cycle. A systematic review. Thromb. Haemost..

[9] Hemker H, Willems G, Beguin S (1986). A computer assisted method to obtain the prothrombin activation velocity in whole plasma independent of thrombin decay processes. Thrombosis and haemostasis.

[10] Chaireti R, Gustafsson KM, Bystrom B, Bremme K, Lindahl TL (2013). Endogenous thrombin potential is higher during the luteal phase than during the follicular phase of a normal menstrual cycle. Hum. Reprod..

[11] Rugeri L (2007). Thrombin-generating capacity in patients with von Willebrand’s disease. Haematologica.

[12] Cederblad G, Hahn L, Korsan-Bengtsen K, Pehrsson N, Rybo G (1977). Variations in blood coagulation, fibrinolysis, platelet function and various plasma proteins during the menstrual cycle. Pathophysiol. Haemost. Thromb..

[13] Dapper DV, Didia BC (2002). Haemorheological changes during the menstrual cycle. East Afr. Med. J..

[14] Koh SC, Prasad R, Fong Y (2005). Hemostatic status and fibrinolytic response potential at different phases of the menstrual cycle. Clinical and applied thrombosis/hemostasis.

[15] Lebech AM, Kjaer A (1989). Lipid metabolism and coagulation during the normal menstrual cycle. Horm. Metab. Res..

[16] Blomback M, Eneroth P, Landgren BM, Lagerstrom M, Anderson O (1992). On the intraindividual and gender variability of haemostatic components. Thromb. Haemost..

[17] Blomback M, Landgren BM, Stiernholm Y, Andersson O (1997). The effect of progesterone on the haemostatic mechanism. Thromb. Haemost..

[18] Önundarson PT, Gudmundsdottir BR, Arnfinnsdottir AV, Kjeld M, Ólafsson Ö (2001). Von Willebrand factor does not vary during normal menstrual cycle. Thromb. Haemost..

[19] Repina MA, Korzo TM, Zinina TA (2002). Effect of hormone replacement therapy with femoston on hemostasis in peri- and postmenopausal women. Med. Sci. Monit..

[20] Siegbahn A, Odlind V, Hedner U, Venge P (1989). Coagulation and fibrinolysis during the normal menstrual cycle. Ups. J. Med. Sci..

[21] Mandalaki T, Louizou C, Dimitriadou C, Symeonidis P (1980). Variations in factor VIII during the menstrual cycle in normal women. N. Engl. J. Med..

[22] Miller CH, Dilley AB, Drews C, Richardson L, Evatt B (2002). Changes in von Willebrand factor and factor VIII levels during the menstrual cycle. Thromb. Haemost..

[23] Govorov I (2016). Heavy menstrual bleeding and health-associated quality of life in women with von Willebrand’s disease. Exp. Ther. Med..

[24] Björk, I. & Olson, S. T. In *Chemistry and biology of serpins* 17–33 (Springer, 1997).

[25] Brummel-Ziedins K, Vossen CY, Rosendaal FR, Umezaki K, Mann KG (2005). The plasma hemostatic proteome: thrombin generation in healthy individuals. J. Thromb. Haemost..

[26] Giardina EG, Chen HJ, Sciacca RR, Rabbani LE (2004). Dynamic variability of hemostatic and fibrinolytic factors in young women. J. Clin. Endocrinol. Metab..

[27] Loeffen R (2012). Preanalytic variables of thrombin generation: towards a standard procedure and validation of the method. J Thromb Haemost.

[28] Moeller A, Weippert-Kretschmer M, Prinz H, Kretschmer V (2001). Influence of ABO blood groups on primary hemostasis. Transfusion.

[29] O’donnell J, Laffan M (2001). The relationship between ABO histo‐blood group, factor VIII and von Willebrand factor. Transfusion Medicine.

